# The study of cardiovascular risk in adolescents – ERICA: rationale, design and sample characteristics of a national survey examining cardiovascular risk factor profile in Brazilian adolescents

**DOI:** 10.1186/s12889-015-1442-x

**Published:** 2015-02-07

**Authors:** Katia Vergetti Bloch, Moyses Szklo, Maria Cristina C Kuschnir, Gabriela de Azevedo Abreu, Laura Augusta Barufaldi, Carlos Henrique Klein, Maurício TL de Vasconcelos, Glória Valéria da Veiga, Valeska C Figueiredo, Adriano Dias, Ana Julia Pantoja Moraes, Ana Luiza Lima Souza, Ana Mayra Andrade de Oliveira, Beatriz D’Argord Schaan, Bruno Mendes Tavares, Cecília Lacroix de Oliveira, Cristiane de Freitas Cunha, Denise Tavares Giannini, Dilson Rodrigues Belfort, Dulce Lopes Barboza Ribas, Eduardo Lima Santos, Elisa Brosina de Leon, Elizabeth Fujimori, Elizabete Regina Araújo Oliveira, Erika da Silva Magliano, Francisco de Assis Guedes Vasconcelos, George Dantas Azevedo, Gisela Soares Brunken, Glauber Monteiro Dias, Heleno R Correa Filho, Maria Inês Monteiro, Isabel Cristina Britto Guimarães, José Rocha Faria Neto, Juliana Souza Oliveira, Kenia Mara B de Carvalho, Luis Gonzaga de Oliveira Gonçalves, Marize M Santos, Pascoal Torres Muniz, Paulo César B Veiga Jardim, Pedro Antônio Muniz Ferreira, Renan Magalhães Montenegro, Ricardo Queiroz Gurgel, Rodrigo Pinheiro Vianna, Sandra Mary Vasconcelos, Sandro Silva da Matta, Stella Maris Seixas Martins, Tamara Beres Lederer Goldberg, Thiago Luiz Nogueira da Silva

**Affiliations:** Universidade Federal do Rio de Janeiro, Instituto de Estudos em Saúde Coletiva, Rio de Janeiro, Brazil; Universidade do Estado do Rio de Janeiro, Núcleo de Estudos da Saúde do Adolescente, Rio de Janeiro, Brazil; Fundação Oswaldo Cruz, Escola Nacional de Saúde Pública, Rio de Janeiro, Brazil; Escola Nacional de Ciências Estatísticas, Fundação Instituto Brasileiro de Geografia e Estatística (ENCE/IBGE), Rio de Janeiro, Brazil; Universidade Federal do Rio de Janeiro, Instituto de Nutrição Josué de Castro, Rio de Janeiro, Brazil; Universidade Estadual Paulista, Faculdade de Medicina, São Paulo, Brazil; Universidade Federal do Pará, Instituto Ciências da Saúde, Pará, Brazil; Universidade Federal de Goiás, Faculdade de Enfermagem e Nutrição, Goiás, Brazil; Universidade Estadual de Feira de Santana, Departamento de Saúde, Bahia, Brazil; Universidade federal do Rio Grande do Sul, Hospital de Clínicas de Porto Alegre, Rio Grande do Sul, Brazil; Universidade Federal do Amazonas, Instituto de Saúde e Biotecnologia, Amazonas, Brazil; Universidade do Estado do Rio de Janeiro, Departamento de Nutrição, Rio de Janeiro, Brazil; Universidade federal de Minas Gerais, Hospital de Clínicas da UFMG, Minas Gerais, Brazil; Universidade do Estado do Rio de Janeiro, Centro Biomédico – Nutrição, Rio de Janeiro, Brazil; Universidade Federal do Amapá, Amapá, Brazil; Universidade Federal do Mato Grosso do Sul, Curso de Nutrição, Mato Grosso do Sul, Brazil; Instituto Federal de Educação Técnico Tecnológico do Tocantins, Tocantis, Brazil; Universidade Federal do Amazonas, Faculdade de Educação Física e Fisioterapia, Amazonas, Brazil; Universidade de São Paulo, Departamento de Enfermagem em Saúde Coletiva, São Paulo, Brazil; Universidade Federal do Espirito Santo, Departamento de Enfermagem, Espírito Santo, Brazil; Universidade Federal de Santa Catarina, Centro de Ciências da Saúde, Departamento de Nutrição, Santa Catarina, Brazil; Universidade Federal do Rio Grande do Norte, Centro de Biociências, Departamento de Morfologia, Rio Grande do Norte, Brazil; Universidade Federal de Mato Grosso, Instituto de Saúde Coletiva, Departamento de Saúde Coletiva, Mato Grosso, Brazil; Instituto Nacional de Cardiologia, Laboratório de Biologia e Diagnósticos Moleculares, Rio de Janeiro, Brazil; Universidade Estadual de Campinas, Faculdade de Ciências Médicas, Departamento de Medicina Preventiva e Social, São Paulo, Brazil; Universidade Estadual de Campinas, Faculdade de Enfermagem, São Paulo, Brazil; Universidade Federal da Bahia, Hospital Ana Neri, Bahia, Brazil; Pontifícia Universidade Católica do Paraná, Escola de Medicina, Paraná, Brazil; Universidade Federal de Pernambuco, Centro Acadêmico de Vitória, Pernambuco, Brazil; Universidade de Brasília, Departamento de Nutrição, Distrito Federal, Brazil; Universidade Federal de Rondônia, Núcleo de Saúde, Departamento de Educação Física, Rondônia, Brazil; Universidade Federal do Piauí, Centro de Ciências da Saúde, Departamento de Nutrição, Piauí, Brazil; Universidade Federal do Acre, Departamento de Ciências da Saúde e Educação Física, Acre, Brazil; Universidade Federal de Goiás, Faculdade de Medicina, Goiás, Brazil; Universidade Federal do Maranhão, Programa de Pós-graduação em Saúde Coletiva, Maranhão, Brazil; Universidade Federal do Ceará, Faculdade de Medicina, Departamento de Saúde Comunitária, Ceará, Brazil; Universidade Federal de Sergipe, Centro de Ciências Biológicas e da Saúde, Departamento de Medicina, Sergipe, Brazil; Universidade Federal da Paraíba, Centro de Ciências da Saúde - Campus I, Departamento de Nutrição, Paraíba, Brazil; Universidade Federal de Alagoas, Departamento de Nutrição, Alagoas, Brazil; Hospital Estadual Getúlio Vargas, Núcleo Hospitalar de Geriatria e Gerontologia, Rio de Janeiro, Brazil; Universidade Federal de Roraima, Centro de Ciências da Saúde, Roraima, Brazil; Universidade Estadual Paulista Júlio de Mesquita Filho, Faculdade de Medicina de Botucatu, Departamento de Pediatria, São Paulo, Brazil

**Keywords:** Cardiovascular diseases, Metabolic syndrome X, Adolescent

## Abstract

**Background:**

The Study of Cardiovascular Risk in Adolescents (Portuguese acronym, “ERICA”) is a multicenter, school-based country-wide cross-sectional study funded by the Brazilian Ministry of Health, which aims at estimating the prevalence of cardiovascular risk factors, including those included in the definition of the metabolic syndrome, in a random sample of adolescents aged 12 to 17 years in Brazilian cities with more than 100,000 inhabitants. Approximately 85,000 students were assessed in public and private schools. Brazil is a continental country with a heterogeneous population of 190 million living in its five main geographic regions (North, Northeast, Midwest, South and Southeast). ERICA is a pioneering study that will assess the prevalence rates of cardiovascular risk factors in Brazilian adolescents using a sample with national and regional representativeness. This paper describes the rationale, design and procedures of ERICA.

**Methods/Design:**

Participants answered a self-administered questionnaire using an electronic device, in order to obtain information on demographic and lifestyle characteristics, including physical activity, smoking, alcohol intake, sleeping hours, common mental disorders and reproductive and oral health. Dietary intake was assessed using a 24-hour dietary recall. Anthropometric measures (weight, height and waist circumference) and blood pressure were also be measured. Blood was collected from a subsample of approximately 44,000 adolescents for measurements of fasting glucose, total cholesterol, HDL-cholesterol, LDL-cholesterol, triglycerides, glycated hemoglobin and fasting insulin.

**Discussion:**

The study findings will be instrumental to the development of public policies aiming at the prevention of obesity, atherosclerotic diseases and diabetes in an adolescent population.

## Background

Cardiovascular diseases (CVD) are the main cause of mortality in Brazil [1] and it is well known that children/adolescents with cardiovascular risk factors have increased atherosclerosis progression rate in adulthood [2].

The prevalence of overweight and obesity is increasing worldwide, affecting all age groups [3] and these conditions in childhood are predictive of obesity in adults [4]. Obesity in children and adolescents is strongly associated with insulin resistance, dyslipidemia and type 2 diabetes [5]. In Brazil, overweight and obesity have increased in all age groups, including children and adolescents [6]. The Household Budget Survey (POF) 2008–2009 showed a striking increase in the prevalence of overweight among adolescents (10 to 19 years of age) from 1974/1975 to 2008/2009. During this period, overweight increased from 3.7% to 21.7% in males and from 7.6% to 19.4% in females [6]. In addition to obesity and dyslipidemia, studies have shown that childhood risk factors such as smoking, elevated blood pressure, low physical activity, and low consumption of fruits and vegetables are predictive of subclinical atherosclerosis, as evaluated by carotid artery intima–media thickness, and its progression in adulthood [7,8]. Besides individual’s risk factors, low socioeconomic situation and parental smoking during childhood are predictors of increased carotid intima–media thickness [9].

What follows is a description of the design and procedures of the Study of Cardiovascular Risk Factors (Portuguese acronym, ERICA).The objective is to estimate the prevalence of cardiovascular risk factors, including diabetes mellitus, obesity, hypertension, dyslipidemia, active and passive smoking, physical inactivity, unhealthy food consumption, and the association between these factors in adolescents aged 12 through 17 years who attend public and private schools in Brazilian cities with more than 100,000 inhabitants. For non-invasive procedures, 85,000 adolescents were studied; in a sub-sample of approximately 44,000 adolescents, blood was collected for measurements of glucose, lipids, and insulinemia.

ERICA is a school-based cross-sectional multi-center study. Its specific aims are (1) to describe population patterns of anthropometric parameters; (2) to describe population patterns of blood pressure (BP) levels; (3) to assess ethnic, age, sex, socioeconomic, and regional differences in CVD risk factors prevalence rates; and (4) to study the association of CVD risk factors with life style characteristics.

## Methods/Design

### Study sample

ERICA’s sample consists of 85,000 adolescents aged 12 to 17 years enrolled in morning or afternoon shifts of private and public schools located in the 273 Brazilian municipalities with more than 100,000 inhabitants in July, 1, 2009. Manuscript describing ERICA’s sample is in press [10].

The population frame that was sampled was stratified into 32 geographic strata formed as follows: each of the 27 federation unit capital; and five strata comprising the municipalities of each of the five macro-regions of the country. After geographic stratification, two successive selection stages were implemented: selection of schools and selection of school classes.

The schools were selected in each geographic stratum with probability proportional to size (PPS). The size measure of each school was set equal to the ratio between the number of students in its eligible classes and the distance from the State capital. This strategy aimed to concentrate the sample around the State capitals, reducing the study’s costs and facilitating the survey logistic, particularly that related to the blood drawing.

The PPS selection was performed in each geographic stratum after sorting the school records by situation (urban or rural areas) and the school governance (private or public). One thousand two hundred and fifty one schools in 124 municipalities were selected.

In the second stage, three classes in each sampled school were selected with equal probabilities during field work. Using class year as a proxy of age, only the classes of 7th, 8th and 9th years of elementary school and 1st, 2nd and 3rd year of high school were eligible for selection.

A spreadsheet was used for class selection. The spreadsheet was specific to each sampled school, with random numbers and preprogrammed formulas for class selection and for the selection of two students who had a repeat 24-hour food dietary recall for evaluation of reliability of the dietary questionnaire.

In each selected class, all students were invited to participate in an exam consisting of interviews and anthropometric and BP measurements. Because fasting was needed for blood drawing, only those students in the morning shift classes were invited for this procedure. Schools’ and parents’ permission were secured.

### Sample size estimation

For the calculation of sample size, a prevalence of the metabolic syndrome in adolescents of 4% was used with a maximum absolute error of 1% and confidence level of 95%. Using these parameters, a simple random sample size would be estimated at 1,475 adolescents. Considering that the sample is clustered by school and class, a design effect of 3 was used (a previous survey with students in Rio de Janeiro indicated a design effect for body mass index of 2.87), yielding a sample size of 4,425 adolescents. As the estimates with controlled precision had to be produced for 12 domains (6 ages × two sexes) it was estimated that the overall sample size would have to be 74,340 individuals.

The sample size allocation among the 32 strata was of a power type, proportional to the square root of the survey population in each stratum, according to the 2009 Brazilian Educational Census (reviewed in 2011) [11].

### Information system

ERICA’s information system consists of four data modules. The first module, ERICA Web, requires internet access and allows performance of the following tasks: (1) registration of schools and students; (2) transfer of data to and from the server; (3) ready access to data; (4) printing of the class check list; and (5) printing of the student’s and school results.

The second module is ERICA PDA (personal digital assistant). It registers the responses to the questionnaire, anthropometric and BP measurements, and the school questionnaire.

The third module is specific for entering data from the 24-hour dietary recall (24hR).

The fourth module is a set of questions about the fasting period; it is applied before blood collection.

One of the major advantages of ERICA’s information system is that all data was immediately available after data collection.

Data storage and management is centralized at Federal University of Rio de Janeiro (Portuguese acronym, UFRJ), using a cloud service. The operational system is Windows Server 2008, the dataset is Firebird 2.5, and the language C#.NET. The software used to develop the 24 hR was Visual Studio NET 2012 (web site and desktop).

### Instruments and procedures

Three questionnaires were applied: one for adolescents, one for parents/care givers, and one about school characteristics.

The questionnaire for students and that about the school were partially based on instruments used in other studies on risk factors in the young in Brazil [12].

#### Adolescent’s questionnaire

This questionnaire was self-administered using a PDA, model LG GM750Q. Its main areas (Table 1) were socioeconomic status, adolescent work, smoking, alcohol consumption, physical activity assessment, health and medical history, sleeping hours, feeding behavior, oral health, common mental disorder, and reproductive health.

**Table 1:** **Information from ERICA’s questionnaires**

**Questionnaire**	**Issue**	**Variable**
**Adolescent**	Sociodemographic characteristics	Age, gender, and race/ethnicity
		Parents’ education
		Household assets
		Number of residents/room
	Adolescent work	Job characteristics
	Physical activity	Type, duration, and frequency of activity [13]
		Active/inactive [14]
	Feeding behavior	Eating with parents
		Breakfast
		Eating at school
		Eating in front of TV
		Snacks
		Drinking water
		Eating fish
		Use of sweeteners
	Smoking	Experimentation
		Initiation/intensity
		Use of flavoured cigarrets
		Smoking exposure
	Alcohol consumption	Experimentation
		Initiation/intensity/type of beverage
	Reproductive health	Sexual secondary characteristics
		Menarche
		Pregnancy
		Use of contraceptive methods
		Sexual maturation self-classification using Tanner’s pictures
	Oral health	Gum bleeding
		Teeth brushing
		Dental floss use
	Medical health history	Hypertension
		Diabetes mellitus
		Hypercholesterolemia
		Ashma
		Bodyperception
	Sleep	Bed time and waketime
	Commom mental disorders	General Health Questionnaire (GHQ12) [15]
**Parents**	Adolescent mother characteristics	Age, race/ethnicity, education Occupation
		Self-reported weight and height
	Family medical and health history	High blood pressure
		Ischemic heart disease
		Cerebral vascular disease
		Diabetes mellitus
		High cholesterol
		Father’s referred weight and height
	Adolescent birth history	Gestational age
		Birth weight/length
		Breastfeeding
		Birth place
	Adolescent medical health history	Diseases
		Hospital admissions, including emergency admissions
	Adolescent sleep	Sleep duration
**School**	School general characteristics	Number of students/classes
		Number of physical education teachers
		Extra-curricular activities (arts, sports)
	Physical structure	Sports court
		Pool
		Auditorium
		Computer lab
		Drinkers
	Eating at school	School meals
		Food sold in school
		Cafeteria
		Food advertising

#### School questionnaire

The school questionnaire was filled out by a field researcher using the PDA. It contained information about school characteristics related to physical structure, availability of physical education teachers, and school meals/sale of food (Table 1).

#### Parents’/caregiver’s questionnaire

Parents’/care giver’s questionnaire provided information on mothers’ educational achievement, family history of cardiovascular and metabolic diseases, and circumstances related to student’s birth (birth weight, breastfeeding). A printed form was sent to the parents’/caregivers’ by the students. This is the only information source that was collected using a hard (printed) form. Data was double entered to avoid typing errors. Table 1 describes the main variables that will be assessed.

#### 24-hour dietary recall

Dietary intake was assessed using a 24hR in a face to face interview that was performed by trained interviewers, using a specific software to register information directly in a netbook using the multiple pass method [16]. In a random subsample of two students per class, the students responded to a second 24hR in order to estimate intra-individual variability. Food and drinks consumed were recorded for all meals and snacks before the interview, with quantities and preparation methods whenever appropriate. Portion size estimation was obtained by showing photographs included in the software. The data was electronically transferred to the central database.

After conversion of the food items to grams [17], the data set will be linked to a nutritional composition table [18] in order to obtain the macro and micronutrient consumption of each adolescent.

The MSM (Multiple Source Method) program [19] will be used to calculate, by the difference between the 1st and the 2nd 24hR, a correction factor for each macro and micronutrient to be applied to all sample. The objective is to remove the intra-individual variability and to allow the estimation of usual intake. The method is applicable to nutrient and food intake including episodically consumed foods.

#### Anthropometric measurements

Trained researchers, according to written standardized procedures, performed the measurements.

##### Height, weight and circumferences

Anthropometric measurements were done with the individuals wearing light clothing and no shoes. Height [20] was measured to the nearest 1 mm using a calibrated stadiometer (portable stadiometer Alturexata®, Minas Gerais, Brazil) with millimeter resolution and height up to 213 cm. The subjects were in full standing position (in the Frankfort horizontal plane). Measurements were made in duplicate for quality control purposes. A maximum variation of 0.5 cm was allowed between the two measurements. The PDA system automatically calculated the average of the two measures for use in the analysis. If the difference exceeded 0.5 cm, the measures were deleted in the PDA display and height was measured again.

Weight [20] was measured to the nearest 50 g using a digital scale (model P150m, 200 kg of capacity and 50 g of precision, Líder®, São Paulo, Brazil).

The body mass index (BMI), defined as weight (Kg) divided by the square of height (meters), was calculated. To determine the weight categories of adolescents, the WHO (World Health Organization) reference curves [21], using the index BMI/age, according to sex, was used. The cutoff points were: malnutrition Z-score < −3; low weight Z-score ≥ −3 and < −1; normal weight Z-score ≥ −1 and ≤ 1; overweight Z-score > 1 and ≤ 2; obesity Z-score > 2.

As an additional estimate of body fat distribution, waist circumference was measured as a surrogate of central adiposity. It was measured to the nearest 1 mm using a fiber glass anthropometric tape, with millimeter resolution and length of 1.5 meters (Sanny®, São Paulo, Brazil). The individuals was at the upright position, with abdomen relaxed at the end of gentle expiration [22]. The measurement was done horizontally, at half the distance between the iliac crest and the lower costal margin. Measurements was done in duplicate for quality control purposes. A maximum variation of 1 cm between the two measurements was allowed. The PDA system automatically calculated the average of the two measures to be used in the analysis. If the difference exceeded this value, the values will be deleted in the PDA display and the waist circumference must be measured again.

Arm length was measured from the acromion (bony extremity of the shoulder girdle) to the olecranon (tip of the elbow) using an anthropometric tape. The midpoint on the dorsal (back) surface of the arm was marked with a pen. The participant was asked to relax the arm alongside the body and the measuring tape will be placed snugly around the arm at the midpoint mark, keeping the tape horizontally [20]. The tape should not indent the skin.

#### Blood pressure evaluation

Blood pressure measurements were based on the 4th Report on the Diagnosis, Evaluation, and Treatment of High Blood Pressure in Children and Adolescents, published in 2004 [23]. Systolic and diastolic BP and pulse were measured using the automatic oscillometric device Omron® 705-IT(Omron Healthcare, Bannockburn, IL, USA). This model has been validated for adolescents [24] and it has been chosen due to its ease of use and the minimization of observer bias or digit preference (which are the common errors associated with the auscultatory method) [25].

The adolescent were told to avoid stimulant drugs or foods, and sit quietly for 5 minutes, with his or her back supported, feet on the floor and right arm supported, and the antecubital fossa at heart level. The appropriate cuff size for the adolescent’s upper right arm was indicated when arm circumference is registered in the PDA. Three consecutive measures were taken for each individual, with an interval of three minutes between each. The 2nd and 3^rd^BP readings were used in order to reduce the impact of reactivity on the blood pressure (higher first reading).

The definition of hypertension in children and adolescents was based on the normative distribution of BP in healthy children. Normal BP was defined as SBP (systolic BP) and DBP (diastolic BP) that were <90th percentile for gender, age, and height. Hypertension was defined as average SBP or DBP that were ≥95th percentile for gender, age, and height. Average SBP or DBP levels that were ≥ 90th percentile but <95^th^percentile were designated as “high normal” and were considered to be an indication of heightened risk for developing hypertension. This designation is consistent with the description of pre-hypertension in adults. It is now recommended that, as with adults, children and adolescents with BP levels ≥ 120/80 mmHg but <95th percentile should be considered prehypertensive [23].

#### Biochemical evaluation

Blood samples were collected from a subsample of approximately 44,000 students by qualified professionals in the participating schools.

This subsample was comprised by all the students studying during the morning who accepted to collect blood and who had written permission of their parents/guardians.

Participants were oriented to keep an overnight fast within12 hours before de exam. Fasting blood samples were collected for analyses of glucose, insulin, lipid profile (total cholesterol, HDL cholesterol and triglycerides), and glycated hemoglobin (HbA1c).The cutoff points that were used for these tests are shown in Table 2. The blood samples were processed and plasma and serum separated within two hours after collection and kept between 4° and 10°C while moved to the study’s single laboratory. Table 2 contains also the description of the analytical methods used for each parameter. In a subsample of 7,800 adolescents from four States, Rio de Janeiro, Rio Grande do Sul, Ceará and Federal District, serum was stored in freezers at −80°C for future analyses.

**Table 2:** **Classification criteria: cutoff points used for blood testing results**

**Exam**	**Method** ^**a**^	**Cutoff points**		
		**Desirable**	**Borderline**	**High**
Colesterol (mg/dL) (mmol/l) [26]	Enzymatic kinetics	<150	150-169	≥170
		<3.9	3.9-4.39	≥4.4
LDL-C (mg/dL) (mmol/l) [26]	Enzymatic colorimetric assay	<100	100-129	≥130
		<2.59	2.59-3.34	≥3.36
HDL-C (mg/dL) (mmol/l) [26]	Enzymatic colorimetric assay	≥45	---	---
		≥1.16		
Triglycerides (mg/dL) (mmol/l) [26]	Enzymatic kinetics	<100	100-129	≥130
		<1.13	1.13-1.46	≥1.47
Glucose (mg/dL) (mmol/l) [27]	Hexoquinase method	70-99	100-125,9	≥126
		3.89-5.5	5.6-6.9	≥7.0
Insulin (mU/L) [28]	Chemiluminescence	<15	15-20	≥20
Glycated hemoglobin [27]	Ion exchange chromatography	<5.7	---	≥5.7

^a^Brazilian Society of Pathology; LDL-C: low density cholesterol; HDL-C: high density cholesterol.

#### Notification of and referral for study findings

The results of the blood tests, anthropometric measurements and BP levels were given directly to each student. In cases where abnormal biochemical, anthropometrical or BP values were identified, according to clinical cut-off points, participants were contacted and referred to health units or to their own physicians when the following results were obtained:

- Undernourishment (<−3 z score).
- Obesity (>2 z score).
- High BP(>95th percentile).
- High total cholesterol or triglycerides levels (>170 mg/dl, >130 mg/dL, respectively).
- Abnormal fasting glycemia (100–125.9 mg/dL) or diabetes mellitus suspicion (≥126 mg/dL).

The adolescent was asked to have a repeated BP measurement within one year when his/her systolic or diastolic BP were ≥90th percentile but <95th or >120/80 and <95th percentile.

The adolescent was promptly referred to a health unit when his/her glucose levels were >200 mmHg or his/her BP levels were > 99th.

### Quality assurance and quality control

A pretest was conducted in a public school in Rio de Janeiro in 2011. This process provided crucial and detailed insight into the understanding of all questionnaire items by the school’s students and also allowed us to analyze logistical issues related to the various stages of the field work.

A pilot study was conducted in five cities (Rio de Janeiro, Cuiabá, Feira de Santana, Botucatu, and Campinas) located in different regions of Brazil, with participation of two public and one private school in each city. Nearly 1,300 adolescents were evaluated in this pilot study pertaining to the non-invasive procedures, and for 600 students in the morning shift, blood samples were collected. The data analysis of the pilot study provided clues about points to be reinforced in the training of supervisors and field work.

To prevent or minimize errors during the data collection, procedures were adopted to ensure the quality of information. An operations manual was prepared with detailed descriptions of the standardized procedures. The field team was carefully trained before the start of the study. Data collection was monitored throughout the study.

Internal and external quality control (QC) was conducted by the study’s laboratory. Internal QC was performed daily to monitor reliability through repeated measurements of the same sample. External QC or proficiency testing was monitored accuracy through comparisons between the study’s laboratory and a gold standard laboratory. During the study, phantom samples was available from the Brazilian Society of Pathology (Control Lab) [29] and the Brazilian Society of Clinical Analysis (National Program of Quality Control) [30] for this purpose.

#### Training

Training of field workers was performed by the ERICA’s central coordination team according to the study protocol. Videos were especially produced for the training of anthropometric and BP measures. Interviewers were trained in anthropometric measurements using the Habicht’s criterion as a guide [31].

A series of logic checks were conducted regularly to identify outliers, discrepancies or digit preference in measurements. Appropriate measures were taken whenever problems were detected. If necessary, examiners were retrained, and equipment was constantly checked and replaced.

Extreme values were those suspected to be unusual, but permitted by the instruments. An alert rule was created so that when more than 10% of the records of an observer were above or below the 5th and 95th percentiles, according to the measurements obtained in the pilot study, a suspected measurement error was flagged, and indicated the need for retraining the observer.

### Study planning and management

The study’s Steering Committee was responsible for planning, overseeing the conduct of the study and applying appropriate corrective measures based on QC results. In each of the 27 Federal (geographic) Units (26 States and the Federal District), local leaders managed the regional data collection. Several researchers collaborated as consultants at different stages of the study. Subcommittees worked to develop tools and strategies for data collection, development of the information system and QC. Members of the Steering Committee developed an operation manual and trained teams of 27 regional leaders and field supervisors. In each state, 3 to 6 teams of supervisors were trained.

A firm with extensive experience in logistics of large scale research was hired to recruit and train the field staff, under the supervision of the Steering Committee and the regional leaders. Each team had five field workers and two supervisors. The same firm was responsible for transporting the equipment between States.

### Statistical analysis

When analyzing the data, the sample weights corresponding to the reciprocal of the product of the inclusion probabilities in each sample stage will be considered. A correction for non-response will be introduced in the weights. The need for sample weight calibration by sex and each age class will also be considered.

Prevalence rates, confidence intervals, and measures of central tendency will be estimated taking into account the sampling weights and the other structural sample design variables (stratum and primary sampling unit-PSU). Expected outcome measures (prevalence rates and mean variances) and differences between groups (age, gender and urban/rural groups) will be calculated with 95% confidence intervals. Sampling error, which could potentially affect the reliability and thus accuracy of the study results, will be estimated.

The association between demographic, anthropometric, metabolic and lifestyle habits will be investigated in unadjusted analysis as well as stratified and multivariate models. Statistical tests will be applied according to the data distribution and homogeneity of variances of the groups under comparison. An important focus of these analyses is the potential heterogeneity of risk relationships across regions. Testing for interaction will thus be undertaken when appropriate.

### Ethical issues

This study was conducted according to the principles of the Helsinki declaration. The Ethical Committee of the Universidade Federal do Rio de Janeiro approved the study in January, 2009. The approval of the Ethical Committee at each of the 26 States and for the Federal District was obtained. Permission to conduct the study was obtained in all State and local Departments of Education and in all schools.

Written informed consent was obtained from each student, and also from their parents for those who are invited to take blood collection (those studying in the morning). When the local ethics committees require informed parental consent even for students who was not taking blood, such consent was required for students to participate in the study.

During the data collection, care was taken to guarantee the student’s privacy and confidentiality, such as when using folding screens for anthropometric measurements.

## Discussion

The development of ERICA’s procedures presented many challenges. This survey was not only large from the sample size viewpoint, but it was also implemented on a huge geographic scale and in often adverse and heterogeneous conditions. Brazil is the fifth-largest country in the world, so studying a nationally representative sample presents enormous logistic challenges. For the management of a study of such magnitude, the development of an information system with maximum agility was critical.

ERICA will estimate the national prevalence of multiple cardiovascular risk factors in adolescents, including BP and biochemical parameters, not evaluated in other national studies, and will also allow the investigation of their reciprocal relationships and with other health determinants. It will, in addition, be possible to investigate interactions between several socio-demographic characteristics, including region of the country, and selected outcomes, such as diabetes mellitus and hypertension.

The storage of frozen blood in three cities will enable the investigation of inflammatory markers and other possible markers related to cardiovascular diseases and the metabolic syndrome.

Several features such as lifestyle habits, physical activity, smoking, alcohol consumption, minor psychiatric disorders and morbidity such as asthma will be assessed through a questionnaire carefully developed by experts. Details about some eating behaviors, self-perception of weight, hours spent in front of screens and reproductive health and oral hygiene will also be analyzed.

Characteristics of the infrastructure of the school, such as the existence of sports courts and number of physical education teachers, will be correlated with characteristics of adolescents and presence of cardiovascular risk factors.

Another line of research to be followed includes information obtained through the parents/care givers’ questionnaire. Information on birth weight and breastfeeding will be analyzed looking for possible associations with obesity, hypertension and altered glucose and lipid metabolism. Obtaining the name of the mother in these questionnaires will enable the use of techniques of probabilistic database linkage to relate the data from ERICA to the data on Live Births - SINASC. This linkage will allow more detailed information about relevant pregnancy characteristics and birth weight.

Participation of children and adolescents in the labor market is still a matter of debate in Brazil. ERICA will provide information for analysis of the impact of adolescent labor in the distribution of cardiovascular risk factors.

Another strength of ERICA is the possibility of developing BP, weight, height, BMI, and waist circumference distributions by sex, age and, height in Brazilian adolescents. Anthropometric measures can also be analyzed in relation to the adolescents’ self-classifications of sexual maturation, using Tanner’s criteria.

ERICA’s results will contribute to the knowledge about risk factors for atherosclerosis in a young Brazilian population, which may be used not only in guiding adolescents and parents with regard to preventive measures but also in supporting the development of effective, evidence-based health policy involving different sectors of society for the prevention and control of risk factors for diabetes and cardiovascular disease in adolescents. In addition, ERICA will facilitate partnerships between Brazilian academic institutions, as well as between the academia and health and educational public services.

## Abbreviations

24hR: 24-hour dietary recall
BMI: Body mass index
BP: Blood pressure
CVD: Cardiovascular diseases
DBP: Diastolic blood pressure
ERICA: Estudo de Riscos Cardiovasculares em Adolescentes (Study of Cardiovascular Risk Factors)
MSM: Multiple Source Method
PDA: Personal digital assistant
POF: Pesquisa de Orçamentos Familiares (Household Budget Survey)
PPS: Proportional to size
QC: Quality control
SBP: Systolic blood pressure
UFRJ: Universidade Federal do Rio de Janeiro (Federal University of Rio de Janeiro)
WHO: World Health Organization
