# Erratum to: The study of cardiovascular risk in adolescents – ERICA: rationale, design and sample characteristics of a national survey examining cardiovascular risk factor profile in Brazilian adolescents

**DOI:** 10.1186/s12889-015-2083-9

**Published:** 2015-09-03

**Authors:** Katia Vergetti Bloch, Moyses Szklo, Maria Cristina C. Kuschnir, Gabriela de Azevedo Abreu, Laura Augusta Barufaldi, Carlos Henrique Klein, Maurício T L de Vasconcelos, Glória Valéria da Veiga, Valeska C Figueiredo, Adriano Dias, Ana Julia Pantoja Moraes, Ana Luiza Lima Sousa, Ana Luiza Vilela Borges, Ana Mayra Andrade de Oliveira, Beatriz D’Agord Schaan, Bruno Mendes Tavares, Cecília Lacroix de Oliveira, Cristiane de Freitas Cunha, Denise Tavares Giannini, Dilson Rodrigues Belfort, Dulce Lopes Barboza Ribas, Eduardo Lima Santos, Elisa Brosina de Leon, Elizabeth Fujimori, Elizabete Regina Araújo Oliveira, Erika da Silva Magliano, Francisco de Assis Guedes Vasconcelos, George Dantas Azevedo, Gisela Soares Brunken, Glauber Monteiro Dias, Heleno R. Correa Filho, Maria Inês Monteiro, Isabel Cristina Britto Guimarães, José Rocha Faria Neto, Juliana Souza Oliveira, Kenia Mara B. de Carvalho, Luis Gonzaga de Oliveira Gonçalves, Marize M Santos, Pascoal Torres Muniz, Paulo César B. Veiga Jardim, Pedro Antônio Muniz Ferreira, Renan Magalhães Montenegro, Ricardo Queiroz Gurgel, Rodrigo Pinheiro Vianna, Sandra Mary Vasconcelos, Sandro Silva da Matta, Stella Maris Seixas Martins, Tamara Beres Lederer Goldberg, Thiago Luiz Nogueira da Silva

**Affiliations:** Universidade Federal do Rio de Janeiro, Instituto de Estudos em Saúde Coletiva, Rio de Janeiro, Brazil; Universidade do Estado do Rio de Janeiro, Núcleo de Estudos da Saúde do Adolescente, Rio de Janeiro, Brazil; Fundação Oswaldo Cruz, Escola Nacional de Saúde Pública, Rio de Janeiro, Brazil; Escola Nacional de Ciências Estatísticas, Fundação Instituto Brasileiro de Geografia e Estatística (ENCE/IBGE), Rio de Janeiro, Brazil; Universidade Federal do Rio de Janeiro, Instituto de Nutrição Josué de Castro, Rio de Janeiro, Brazil; Universidade Estadual Paulista, Faculdade de Medicina, São Paulo, Brazil; Universidade Federal do Pará, Instituto Ciências da Saúde, Pará, Brazil; Universidade Federal de Goiás, Faculdade de Enfermagem e Nutrição, Goiás, Brazil; Universidade Estadual de Feira de Santana, Departamento de Saúde, Bahia, Brazil; Universidade Federal do Rio Grande do Sul, Hospital de Clínicas de Porto Alegre, Rio Grande do Sul, Brazil; Universidade Federal do Amazonas, Instituto de Saúde e Biotecnologia, Amazonas, Brazil; Departamento de Nutrição, Universidade do Estado do Rio de Janeiro, Rio de Janeiro, Brazil; Universidade Federal de Minas Gerais, Hospital de Clínicas da UFMG, Minas Gerais, Brazil; Universidade do Estado do Rio de Janeiro, Centro Biomédico – Nutrição, Rio de Janeiro, Brazil; Universidade Federal do Amapá, Amapá, Brazil; Universidade Federal do Mato Grosso do Sul, Curso de Nutrição, Mato Grosso do Sul, Brazil; Instituto Federal de Educação Técnico Tecnológico do Tocantins, Tocantis, Brazil; Universidade Federal do Amazonas, Faculdade de Educação Física e Fisioterapia, Amazonas, Brazil; Departamento de Enfermagem em Saúde Coletiva, Universidade de São Paulo, São Paulo, Brazil; Departamento de Enfermagem, Universidade Federal do Espirito Santo, Espírito Santo, Brazil; Departamento de Nutrição, Universidade Federal de Santa Catarina, Centro de Ciências da Saúde, Santa Catarina, Brazil; Departamento de Morfologia, Universidade Federal do Rio Grande do Norte, Centro de Biociências, Rio Grande do Norte, Brazil; Departamento de Saúde Coletiva, Universidade Federal de Mato Grosso, Instituto de Saúde Coletiva, Mato Grosso, Brazil; Laboratório de Biologia e Diagnósticos Moleculares, Instituto Nacional de Cardiologia, Rio de Janeiro, Brazil; Departamento de Medicina Preventiva e Social, Universidade Estadual de Campinas, Faculdade de Ciências Médicas, São Paulo, Brazil; Universidade Estadual de Campinas, Faculdade de Enfermagem, São Paulo, Brazil; Universidade Federal da Bahia, Hospital Ana Neri, Bahia, Brazil; Pontifícia Universidade Católica do Paraná, Escola de Medicina, Paraná, Brazil; Universidade Federal de Pernambuco, Centro Acadêmico de Vitória, Pernambuco, Brazil; Departamento de Nutrição, Universidade de Brasília, Distrito Federal, Brazil; Departamento de Educação Física, Universidade Federal de Rondônia, Núcleo de Saúde, Rondônia, Brazil; Departamento de Nutrição, Universidade Federal do Piauí, Centro de Ciências da Saúde, Piauí, Brazil; Departamento de Ciências da Saúde e Educação Física, Universidade Federal do Acre, Acre, Brazil; Universidade Federal de Goiás, Faculdade de Medicina, Goiás, Brazil; Universidade Federal do Maranhão, Programa de Pós-graduação em Saúde Coletiva, Maranhão, Brazil; Departamento de Saúde Comunitária, Universidade Federal do Ceará, Faculdade de Medicina, Ceará, Brazil; Departamento de Medicina, Universidade Federal de Sergipe, Centro de Ciências Biológicas e da Saúde, Sergipe, Brazil; Departamento de Nutrição, Universidade Federal da Paraíba, Centro de Ciências da Saúde - Campus I, Paraíba, Brazil; Departamento de Nutrição, Universidade Federal de Alagoas, Alagoas, Brazil; Hospital Estadual Getúlio Vargas, Núcleo Hospitalar de Geriatria e Gerontologia, Rio de Janeiro, Brazil; Universidade Federal de Roraima, Centro de Ciências da Saúde, Roraima, Brazil; Departamento de Pediatria, Universidade Estadual Paulista Júlio de Mesquita Filho, Faculdade de Medicina de Botucatu, São Paulo, Brazil

## Text

Here we briefly present an update of the authorship list. After the publication of this study protocol [1], we became aware of the fact that the authors list was incomplete. This may have happened due to the large number of authors and also because some authors have the same first name. The list is now complete and some mistakes in co-authors names and affiliations were corrected. The updated Authors’ Contributions is as follows: KVB, MS, MCCK, CHK, MTLV, GAA, LAB, GVV and VCF have made substantial contributions to the conception and design of the study, drafted the manuscript and revised it critically. AD, AJPM, ALVB, ALLS, AMAO, BDS, CLO, CFC, DTG, DRB, DLBR, ELS, EBL, EF, ERAO, FAGV, GDA, GSB, GMD, HRCF, MIM, ICBG, JRFN, JSO, KMBC, LGOG, MMS, PTM, PCBVJ, PAMF, RMMJr, RQG, RPV, SMV, SSM, SMSM, TBLG, BMT, ESM and TLNS contributed to the conception and design of the study, and revised the manuscript. All authors read and approved the final manuscript. We apologize for all the inconvenience this correction may cause to the readers.
